# Does the skin of mildly hyperthermic individuals display local variations in thermosensitivity for the control of skin blood flow?

**DOI:** 10.1186/2046-7648-4-S1-A94

**Published:** 2015-09-14

**Authors:** Catriona A Burdon, Kyoko Tagami, Joonhee Park, Joanne N Caldwell, Nigel AS Taylor

**Affiliations:** 1 Centre for Human and Applied Physiology, School of Medicine, University of Wollongong, Wollongong, Australia

## Introduction

In an accompanying communication, it was revealed that local variations in cutaneous thermosensitivity, with respect to the control of skin blood flow, were not evident in normothermic individuals. Previously, greater thermosensitivity of the face relative to other sites, including the hand and thigh, was observed for sudomotor control in mildly hyperthermic individuals. Therefore, the possibility was tested that such variations may also exist for vasomotor control when subjects were first rendered mildly hyperthermic.

## Methods

Seven subjects (4 females and 3 males) participated in two trials. Hand and forearm blood flows (right side) were measured in separate trials (water-displacement plethysmography). Deep-body (aural) and skin temperatures were elevated and clamped (whole-body, water-perfusion suit), and then three skin sites (face, hand, thigh; left side) were thermally stimulated (water-perfusion patches). Local skin temperatures were elevated and reduced ~5°C from baseline. Vasomotor sensitivity was calculated for each stimulation from the change in segmental blood flow divided by the change in skin temperature at each treated site.

## Results

Mean body temperature was successfully clamped, averaging 37.8 °C (SD 0.9), and did not differ among trials (*P *> 0.05). These stimulations produced significant and concordant changes in blood flow. A greater thermal stimulus was achieved when cooling (-5.0 °C, SD 0.7) than when heating (4.2 °C, SD 0.6; *P*<0.05), therefore changes in vasomotor sensitivity were used to compare these treatments. Thermosensitivities of the face and hand, with respect to both the hand and forearm vasomotor responses, were greater than those of the thigh during both the local heating and cooling treatments (*P*<0.05). Since there were no differences in the magnitude of the hand and forearm responses to these thermal stimuli (*P *> 0.05), these data were combined, and the resulting outcomes are presented in Table 1.

**Table 1:** Vasomotor sensitivity of the hand and forearm (combined) during three local thermal stimuli.

Treated site	Hand and forearm vasomotor sensitivity (mL.100 mL tissue^-1^.°C^-1^.min^-1^)	
	**Heating**	**Cooling**

Face	1.04 (SD 0.55)*	0.64 (SD 0.50)*

Hand	1.28 (SD 0.82)*	0.63 (SD 0.42)*

Thigh	0.59 (SD 0.29)	0.50 (SD 0.43)

* Significant differences in thermosensitivity relative to the thigh treatment (*P *< 0.05).

## Discussion

Consistent with previous sudomotor research, the face, and now also the hand, displayed greater cutaneous thermosensitivity in mildly hyperthermic subjects. These vascular responses are consistent with the representation of each treated site within the sensory cortex (homunculus), and this may also relate to local variations in thermoreceptor density. The fact that this pattern was not apparent within normothermic individuals (accompanying communication) can be explained by the fact that, in that state, vasomotor drive is dominated by the thermal status of the deep-body tissues. Thus, the centrally mediated vasoconstrictor tone was neither over-ridden nor enhanced by these localised thermal stimuli.

